# Genome sequencing, annotation and exploration of the SO_2_-tolerant non-conventional yeast *Saccharomycodes ludwigii*

**DOI:** 10.1186/s12864-021-07438-z

**Published:** 2021-02-23

**Authors:** Maria J. Tavares, Ulrich Güldener, Ana Mendes-Ferreira, Nuno P. Mira

**Affiliations:** 1 Department of Bioengineering, iBB- Institute for Bioengineering and Biosciences, Instituto Superior Técnico, Universidade de Lisboa, Avenida Rovisco Pais, 1049-001 Lisbon, Portugal; 2 Department of Bioinformatics, Wissenschaftszentrum Weihenstephan, Technische Universität München, Maximus von-Imhof- Forum 3, 85354 Freising, Germany; 3 WM&B – Laboratory of Wine Microbiology & Biotechnology, Department of Biology and Environment, University of Trás-os-Montes and Alto Douro, 5001-801 Vila Real, Portugal; 4 BioISI – Biosystems and Integrative Sciences Institute, Faculdade de Ciências, Universidade de Lisboa, Campo Grande, 1749-016 Lisbon, Portugal

**Keywords:** *Saccharomycodes ludwigii*, *Saccharomycodeacea*, Non-*Saccharomyces* wine yeast, Sulfur resistance, Genome sequencing

## Abstract

**Background:**

*Saccharomycodes ludwigii* belongs to the poorly characterized *Saccharomycodeacea* family and is known by its ability to spoil wines, a trait mostly attributable to its high tolerance to sulfur dioxide (SO_2_). To improve knowledge about *Saccharomycodeacea* our group determined whole-genome sequences of *Hanseniaspora guilliermondii* (UTAD222) and *S. ludwigii* (UTAD17), two members of this family. While in the case of *H. guilliermondii* the genomic information elucidated crucial aspects concerning the physiology of this species in the context of wine fermentation, the draft sequence obtained for *S. ludwigii* was distributed by more than 1000 contigs complicating extraction of biologically relevant information. In this work we describe the results obtained upon resequencing of *S. ludwigii* UTAD17 genome using PacBio as well as the insights gathered from the exploration of the annotation performed over the assembled genome.

**Results:**

Resequencing of *S. ludwigii* UTAD17 genome with PacBio resulted in 20 contigs totaling 13 Mb of assembled DNA and corresponding to 95% of the DNA harbored by this strain. Annotation of the assembled UTAD17 genome predicts 4644 protein-encoding genes. Comparative analysis of the predicted *S. ludwigii* ORFeome with those encoded by other *Saccharomycodeacea* led to the identification of 213 proteins only found in this species. Among these were six enzymes required for catabolism of N-acetylglucosamine, four cell wall β-mannosyltransferases, several flocculins and three acetoin reductases. Different from its sister *Hanseniaspora* species, neoglucogenesis, glyoxylate cycle and thiamine biosynthetic pathways are functional in *S. ludwigii*. Four efflux pumps similar to the Ssu1 sulfite exporter, as well as robust orthologues for 65% of the *S. cerevisiae* SO_2_-tolerance genes, were identified in *S. ludwigii* genome.

**Conclusions:**

This work provides the first genome-wide picture of a *S. ludwigii* strain representing a step forward for a better understanding of the physiology and genetics of this species and of the *Saccharomycodeacea* family. The release of this genomic sequence and of the information extracted from it can contribute to guide the design of better wine preservation strategies to counteract spoilage prompted by *S. ludwigii*. It will also accelerate the exploration of this species as a cell factory, specially in production of fermented beverages where the use of Non-*Saccharomyces* species (including spoilage species) is booming.

**Supplementary Information:**

The online version contains supplementary material available at 10.1186/s12864-021-07438-z.

## Background

*Saccharomycodes ludwigii* is a budding yeast belonging to the *Saccharomycodeacea* family [1], a sister family of the better studied *Saccharomycetacea* family that, among others, includes the paradigmatic species *Saccharomyces cerevisiae*. *S. ludwigii* cells are mostly known for their large-apiculate morphology and spoilage activity over wines (as reviewed by Vejarano et al. [2]). Besides *Saccharomycodes*, the *Saccharomycodeacea* family includes the sister genus *Hanseniaspora, also* harboring species frequently isolated from the “wine environment” like *H. guilliermondii*, *H. uvarum* or *H. opuntiae* [1]. However, while *S. ludwigii* is still seen as a spoilage species, the presence of *H. guilliermondii* and *H. uvarum* has recently been considered positive because these species improve wine aromatic properties by producing aroma compounds that are not produced (or that are produced in very low amounts) by *S. cerevisiae*, the species that leads vinification [3, 4]. Sulfite-preserved grape musts are the niche where isolation of *S. ludwigii* strains is more frequent, although strains have also been isolated at the end of vinification and during storage [1, 5–7]. Several sources have been suggested to serve as reservoirs of *S. ludwigii* including the surface of grapes [8, 9], non-sanitized corks [2, 8, 10] and even cellar equipments [2, 10, 11] thus rendering the control of spoilage prompted by this species difficult. The identification of *S. ludwigii* in plant fluids [8, 11] as well as in the intestinal microbiota of insects found in vineyards [12, 13], led to the hypothesis that these yeasts could be transported from trees to grapes and/or to cellar equipments. This issue, however, still requires further clarification as more information about the species are gathered. The deleterious effects of *S. ludwigii* spoilage in wines are mostly reflected by the high production of off-flavour compunds like acetoin, ethyl acetate, acetaldehyde or acetic acid [2, 5, 10]. Increased formation of sediments or cloudiness are other described effects associated with wine contamination by *S. ludwigii* during bottling and/or storage phases [10, 14]. Besides contaminated wines, *S. ludwigii* strains have also been isolated from other sources such as spoiled carbonated beverages [15], fermented fruit juices [16, 17] or beverages with high ethanol such as mezcal or tequila [18].

The spoilage capacity of *S. ludwigii* to contaminate wines results in great extent from its high tolerance to sulfur dioxide (SO_2_) which is largely used by winemakers as a preservative. Like other organic acids that are also explored as preservatives, the antimicrobial potential of this inorganic acid is dependent on the concentration of the undissociated form (generally designated as “molecular SO_2_”), that predominates at pHs below 1.8 (corresponding to the first pKa of the acid) [19, 20]. At the pH of wine (between 3 and 4) bisulfite (HSO_3_^−^; pKa 6.9) is the most abundant form. After crossing the microbial plasma membrane by simple diffusion, the lipophilic molecular SO_2_ dissociates in the near-neutral cytosol resulting in the release of protons and of bisulfite which, due to its negative charge, cannot cross the plasma membane and accumulates internally [19, 20]. Notably, the accumulation inside *S. ludwigii* cells (at pH 4.0) was significantly lower than the one registered for *S. cerevisiae* [19] that is much more susceptible to SO_2_. That different accumulation was hypothesized (but not experimentally demonstrated) to result from the different lipid composition of the plasma membrane of these two yeasts that may result in different permeabilities to SO_2_ [19]. In the presence of SO_2_ *S. ludwigii* cells excrete high amounts of the SO_2_-sequestering molecule acetaldehyde, however this response does not seem to account for the enhanced tolerance of this species since similar excretion rates were observed in susceptible *S. cerevisae* strains [19]. To counter-act the deleterious effect of intracellular accumulation of SO_2_, *S. cerevisiae* relies on the activity of the sulfite plasma membrane transporter Ssu1, which is believed to promote the extrusion of metabisulfite [21, 22]. The high tolerance to SO_2_ of *Brettanomyces bruxellensis*, another relevant wine spoilage species, was also associated to the activity of Ssu1 [23], however, in *S. ludwigii* no such similar transporter has been described until thus far. In fact, the molecular traits underlying the high tolerance to SO_2_ of *S. ludwigii* remain largely unchacterized.

Recently our group has published the first draft genome of a *S. ludwigii* strain, UTAD17, isolated from a wine must obtained from the demarcated Douro region, in Portugal [24]. However that sequence was scattered across 1360 contigs rendering difficult to have an accurate picture of the genomic portrait of this strain and a realiable extraction of biologically relevant information about *S. ludwigii*. To improve this, the genome of the UTAD17 strain was resequenced using PacBio, resulting in a genome assembled in only 20 contigs and a predicted ORFeome of 4528 canonical protein-coding genes, closer to what is reported for other members of the *Saccharomycodaceae* family (e.g. *H. osmophila*, the species more closely related to *S. ludwigii* that has an annotated genomic sequence encodes 4657 predicted proteins) [25]. This work describes the information extracted from this more refined genomic sequence of the UTAD17 strain shedding light into the biology and physiology of the *S. ludwigii* species with emphasis on the “SO_2_ tolerance” phenotype. Not only this is expected to contribute for the design of better preservation strategies by the wine industry to circumvent spoilage caused by *S. ludwigii*, but this is also expected to accelerate the exploration of this species (and specially of this strain) in production of fermented beverages and in other biotechnological applications. In fact, there is a growing interest of using Non-conventional yeast species, including species previously seen as spoilage, to improve the aroma profile of these beverages and this portfolio of new potentially interesting species includes *S. ludwigii* [26–29].

## Results and discussion

### Overview on the genomic sequence of *S. ludwigii* UTAD17 and on the corresponding functional annotation

**Fig. 1:**
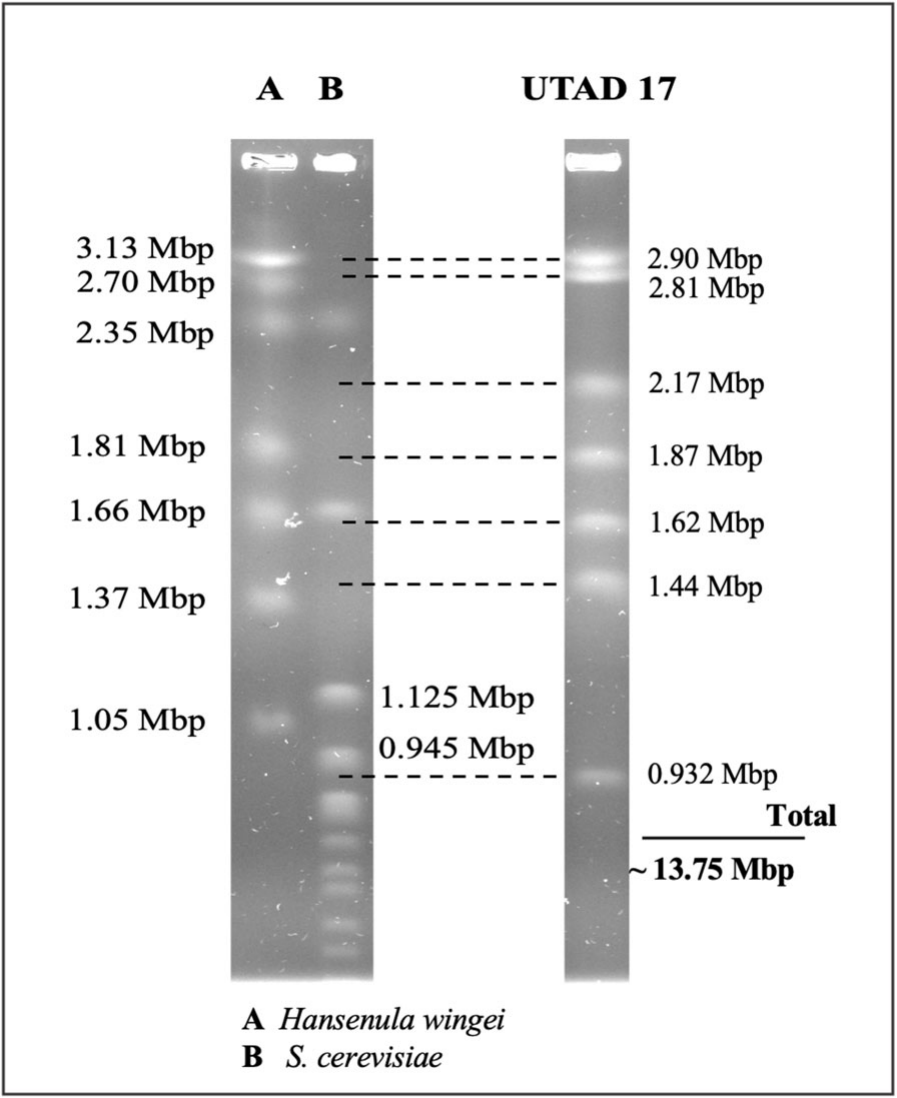
Karyotyping of *Saccharomycodes ludwigii* UTAD17, based on PFGE. Total DNA of *S. ludwigii* UTAD17 was separated by PFGE, as detailed in materials and methods. In the end of the run 7 clearly separated bands, presumed to correspond to the 7 chromosomes of *S. ludwigii* UTAD17, were obtained. Molecular sizes of these chromosomes was estimated based on the migration pattern obtained for the chromosomal bands from *Hansenula wingei* (lane A) and *Saccharomyces cerevisiae* BY4741 that were used as markers  (lane B).

In order to have a suitable portrait of the genomic architecture of the *S. ludwigii* UTAD17 strain karyotyping, based on PFGE, was performed (Fig. 1). The results obtained revealed seven clearly separated chromosomal bands, ranging from 0.9 Mbp to 2.9 Mb, totaling 13.75 Mbp (Fig. 1). This number of chromosomes and their size range is consistent with what was previously described for other *S. ludwigii* strains [30] and is also in line with what is reported for other members of the *Saccharomycodeacea* family [31–33]. Sequencing with PacBio generated 585,118 reads (with a 445.3 coverage) which were de novo assembled into 20 contigs (with sizes ranging from 8.5 kbp to 2.7 Mbp, see supplementary Table S2) and an assembled genome of 12,999,941 bp, corresponding to approximately 95% of the estimated genome size for UTAD17. The genomic properties of *S. ludwigii* UTAD17 are briefly summarized in Table 1, being the features obtained in line with those described for other *Saccharomycodeacea* species [25, 33].

**Table 1:** General features of *S. ludwigii* UTAD17 genome after sequencing and assembly

***S. ludwigii*** UTAD17 genomic features	
**Genome assembly statistics**	
Total number of reads	585,118
Nr. of contigs	20
Coverage	445.3
N50 (bp)	1.48
Maximum contig length (Mbp)	2.7
Minimum contig length (Mbp)	0.9
Average contig length (Mbp)	0.65
Assembly size (Mbp)	13
Average GC content (%)	31

Using the gathered genomic information from *S. ludwigii* UTAD17, in silico annotation was performed exploring results provided by different algorithms used for ab initio gene detection, afterwards subjected to an exhaustive manual curation. Using this approach 5033 protein-encoding genes (CDS) were predicted in the genome of *S. ludwigii* UTAD17, out of which 4644 are believed to encode canonical protein-encoding genes and 389 were considered putative genes since upon BLAST against the UNIPROT database no hit was found (details are provided in supplementary Table S1). The herein described set of *S. ludwigii* proteins represents an increase in the ORFeome of 633 genes (including the 389 considered hypothetical) that had not been disclosed in the initial annotation of the genome of the UTAD17 strain (details provided in supplementary Table S1) [24]. The putative CDSs were distributed throghout 17 of the 20 assembled contigs with genes not being detected only in contigs 14, 16 and 19 (supplementary Table S2). Contigs 14 and 19 share high similarity (above 95% at the nucleotide level) with described mitochondrial DNA from other *S. ludwigii* strains, for which we anticipate these correspond to portions of UTAD17 mitochondrial DNA.

**Fig. 2:**
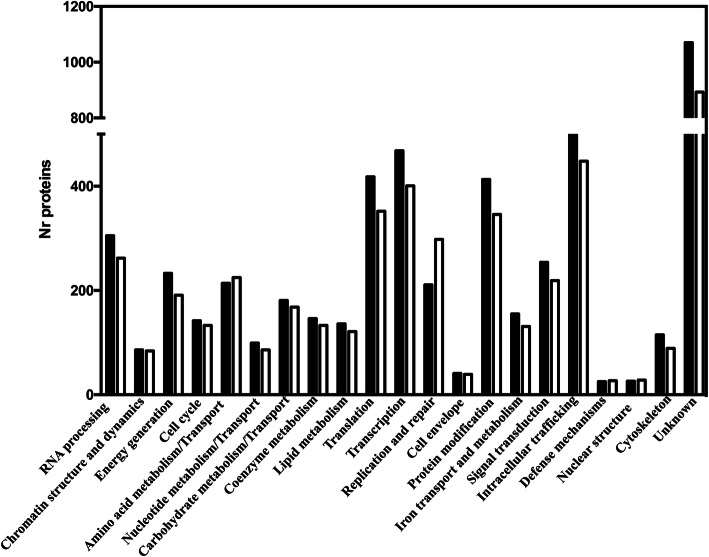
Functional categorization of the predicted ORFeome of *S. ludwigii* UTAD17. After annotation of the assembled genomic sequence, the validated gene models were clustered according with the biological function they are predicted to be involved in (using COG functional categories) using the eggNOG-mapper tool (black bars). As a comparison, the distribution of the *S. cerevisiae* proteome is also shown (white bars). Further details about the functional clustering can be found in supplementary Table S3

To get a more functional view of the *S. ludwigii* UTAD17 ORFeome all the predicted proteins were organized into biological functions using for that the eggNOG-mapper, a tool that enables functional annotation using COG categories [34] (Fig. 2). The highest number of proteins for which it was possible to assign a biological function were clustered in the “Intracellular traficking”, “Transcription”, “Translation” and “Post-translational modification” classes (Fig. 2 and supplementary Table S3), which is consistent with the distribution obtained for *Hanseniaspora* species and also for *S. cerevisiae* (Fig. 2). The number of *S. cerevisiae* genes clustered in 12 of the 21 functional COG classes surpassed those of *S. ludwigii* UTAD17 by approximately 20% (details provided in supplementary Table S3), an observation that is consistent with the later species being pre-whole genome duplication like the other species of the *Saccharomycodeacea* family [1, 33, 35]. Indeed, further mining of *S. ludwigii* UTAD17 genome revealed traits found in pre-whole genome duplication species such as disassembly of the genes necessary for allantoine metabolism, absence of galactose catabolism genes and the lack of a functional pathway for de novo nicotinic acid biosynthesis [35]. Furthermore, out of the 555 ohnologue pairs identified in *S. cerevisiae* [36] we could identify homologues for 517 in the genome of *S. ludwigii* UTAD17, with 512 of these existing in single-copy (that is, the two ohnologues were similar to the same *S. ludwigii* UTAD17 protein) (details in supplementary Table S4).

### Comparative analysis of the predicted proteomes of *S. ludiwgii* with members of the *Saccharomycetaceae* and *Saccharomycodeacea* families

**Fig. 3:**
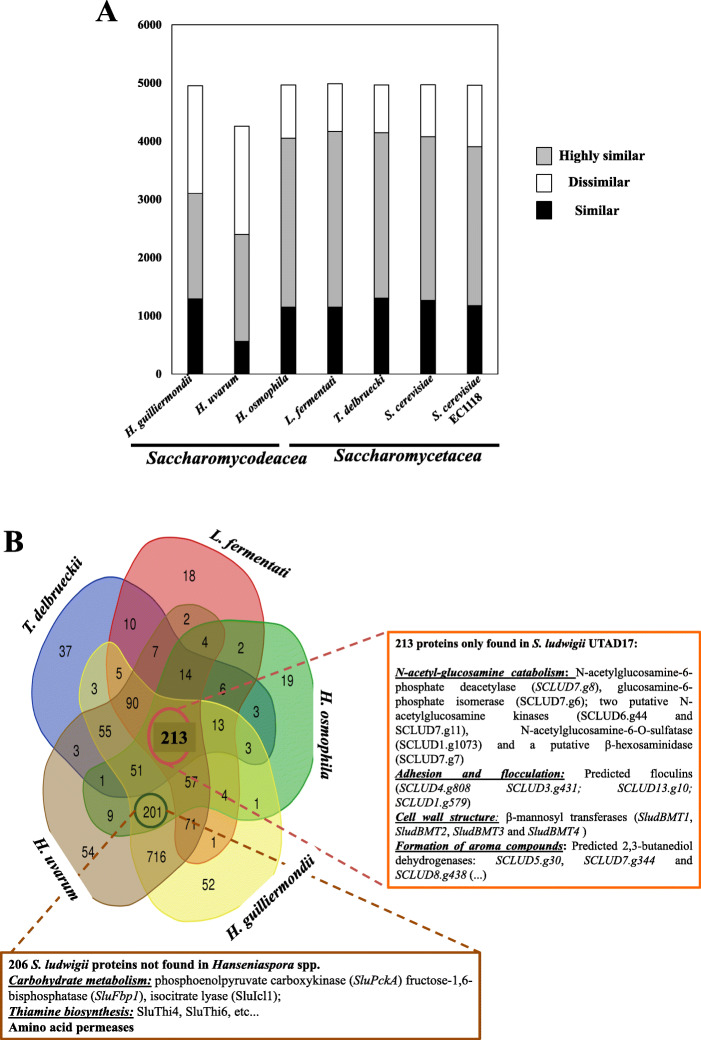
**a** Comparative analysis of the predicted proteome of the Saccharomycodeacea species S. ludwigii, H. guilliermondii, *H. uvarum* and H. *osmophila*. The ORFeome predicted for *S. ludwigii* UTAD17 strain was compared with the one of the *Hanseniaspora* species that also belong to the *Saccharomycodeacea* family using pair-wise BLASTP alignments. Three species belonging to the *Saccharomycetacea* family with relevance in the wine environment, *S. cerevisiae*, *L. fermentati* and *T. delbrueckii* were also included in this comparative analysis. The graph shows the number of *S. ludwigii* proteins highly similar (*e*-value below or equal to e^− 20^ and identity above 50%), similar (e-value below or equal to e^− 20^ and identity between 30 and 50%) or dissimilar (e-value above e^− 20^) from those found in the other yeast species considered. **b** The *S. ludwigii* UTAD17 proteins found to be dissimilar from those found in the other yeast species were compared and the results are shown in the Venn plot. In the picture are highlighted the 526 proteins that were unique of *S. ludwigii* as no robust homologue could be found in any of the other yeast species considered and also the 201 *S. ludwigii* that were found in the *Saccharomycetacea* species but in the other *Saccharomycodeacea* species. Some of the functions represented in these two protein datasets are highlighted in this picture, with the complete list being provided in supplementary Table S5

The get further hints into the physiology of *S. ludwigii* the predicted ORFeome of the UTAD17 strain was compared with the one predicted for *H. guilliermondii*, *H. uvarum* and *H. osmophila*, these representing three species of the *Saccharomycodeacea* family with an available annotated genomic sequence. Three *Saccharomycetacea* species with relevance in the wine environment were also included in this comparative analysis: *Lachancea fermentati*, *Torulaspora delbrueckii* and the *S. cerevisiae* wine strain EC1118 (Fig. 3). The *S. ludwigii* UTAD17 ORFeome showed the highest degree of similarity with *L. fermentati*, *T. delbrueckii* and *H. osmophila*, while similarity with the predicted proteomes of *H. uvarum* and *H. guilliermondii* was considerably smaller (Fig. 3 panel A). This observation was surprising but somehow also in line with the results obtained by phylogenetic analysis of the the ITS sequence of the strains used in this comparative proteomic analysis that also shows a higher divergence of *H. guilliermondii* and *H. uvarum* species within the *Saccharomycodeacea* family (supplementary Figure S1). *H. osmophila* was described to have phenotypic traits similar to those exhibited by *S. ludwigii*, including the ability to survive in high sugar grape musts or reasonable fermentative capacity [6, 37], two traits not associated with *H. uvarum* or *H. guilliermondii.* Similarly, *L. fermentati,* formerly described as *Zygosaccharomyces fermentati* [38], also shares phenotypic traits with *S. ludwigii* including tolerance to SO_2_ and ethanol and the ability to grow on grape-musts or wines with high residual sugar content [39]. Thus, it is possible that the observed higher similarity of the proteomes of *S. ludwigii* with *H. osmophila*, *L. fermentati* and *T. delbrueckii* can result from the evolution of similar adaptive responses to the challenging environment of wine musts, not reflecting their phylogenetic relatedness. In this context, it is intriguing why *H. guilliermondii* and *H. uvarum* are apparently so divergent considering they are also present in grape musts.

**Fig. 4:**
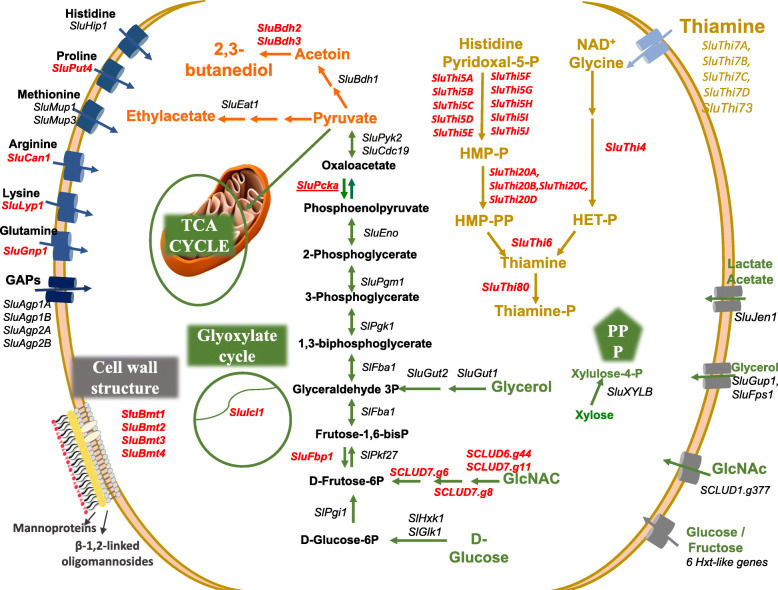
Schematic overview on the central carbon and nitrogen metabolic networks of *S. ludwigii* UTAD17. The predicted ORFeome of *S. ludwigii* was used as an input in the metabolic networks reconstruction tools eggNOG-mapper and KEEG Koala to gather a schematic representation of the metabolic pathways linked to central carbon and nitrogen metabolism active in *S. ludwigii* UTAD17. The picture schematically represents some of the active pathways identified in this in silico analysis, emphasizing in red proteins that were found in *S. ludwigii* but in other *Saccharomycodeacea*. Further information about other proteins also involved in the carbon and nitrogen metabolic networks of *S. ludwigii* are available in supplementary Table S6. This schematic representation is original and was specifically prepared by the authors to be presented in this manuscript

To capture more specific features of the *S. ludwigii* species, the proteins considered dissimilar from those found in the four yeast species used for the comparative proteomic analysis were compared resulting in the Venn plot depicted in Fig. 3 panel B. This analysis identified 213 proteins that were only found in *S. ludwigii* (detailed in supplementary Table S5). This set of proteins included six enzymes required for catabolism of N-acetylglucosamine (GlcNAc) into fructose 6-phosphate including a N-acetylglucosamine-6-phosphate deacetylase (*SCLUD7.g8*), a glucosamine-6-phosphate isomerase (*SCLUD7.g6*) and two putative N-acetylglucosamine kinases (*SCLUD6.g44* and *SCLUD7.g11*) (Fig. 3 panel B, Fig. 4 and supplementary Table S5). A predicted N-acetylglucosamine permease (*SCLUD1.g377*) was also identified in the genome of *S. ludwigii* UTAD17, however, this was also present in the genome of the other four yeast species considered. The set of *S. ludwigii* specific proteins also included a protein weakly similar to a described bacterial N-acetylglucosamine-6-O-sulfatase (*SCLUD1.g1073*) and a putative β-hexosaminidase (*SCLUD7.g7*), these two enzymes being required for catabolism of polysaccharydes harboring GlcNAc like heparine sulphate (Fig. 3 panel B and supplementary Table S3). In yeasts GlcNAc metabolism has been essentially described in dimorphic species like *Candida albicans* or *Yarrowia lypolytica*, where it serves as a potent inducer of morphological transition [40]. Recently, the ability of *Scheffersomyces stipitis* to consume GlcNAc was described enlarging the panoply of GlcNAc consuming yeasts to non-dimorphic species [41]. It is unclear the reasons why GlcNAc catabolism is present in *S. ludwigii* (but not in the other *Saccharomycodeacea*) since there are no reports of this species being dimorphic and we could also not confirm this in the UTAD17 strain (supplementary figure S2). N-acetylglucosamine is a main component of the cell wall of bacteria and fungi, also being present in mannoproteins found at the surface of yeasts cells [42]. In this sense, the ability of *S. ludwigii* to use GlcNAc as a carbon source will likely provide an important advantage in the competitive environment of wine musts in which a strong competition for available sugar takes place. A set of proteins with a predicted function in adhesion and flocculation also emerged among the set of *S. ludwigii*-specific proteins (Fig. 3 panel B). The ability of *S. ludwigii* to cause cloudiness in bottled wines has been described as well as its ability to grow on biofilms [10] or to flocculate even when growing in synthetic growth medium [43]. Further investigations should focus on what could be the role played by these flocullins/adhesins in the aggregation and ability of *S. ludwigii* to form biofilms considering that they are considerably different from the flocullins/adhesins found in the closely related yeast species. A particularly interesting aspect will be to investigate whether these adhesins mediate *S. ludwigii* adherence to the abiotic surfaces of cellars or of cellar equipment.

### Metabolic reconstruction of *S. ludwigii* UTAD17

To reconstruct the *S. ludwigii* metabolic network, the ORFeome predicted for this strain was used as an input for the Koala BLASTX tool [44] resulting in the schematic representation shown in Fig. 4 (the corresponding functional distribution is shown in supplementary Figure S3 while in Supplementary Table S6 are provided further details about the genes clustered in each of the metabolic pathways). This analysis shows that *S. ludwigii* UTAD17 is equipped with all the genes of the main pathways of central metabolism including the pentose phosphate pathway, glycolysis, gluconeogenesis, Krebs cycle and oxidative phosphorylation, besides the already discussed capacity to use GlcNAc (Fig. 4; the identity of enzymes associated to the different enzymatic steps shown in the metabolic map are provided in supplementary Table S6). The fact that *S. ludwigii* UTAD17 is equipped with neoglucogenic enzymes, with an isocitrate lyase and with all the enzymes required for biosynthesis of thiamine is a marked difference from what is observed in other *Saccharomycodeacea* [33] (Fig. 4; Fig. 3 panel B and supplementary Table S6). Considering the critical role of thiamine in driving fermentation, the fact that *S. ludwigii* cells are able to biosynthesize it can be responsible for the higher fermentative capacity of these cells, compared with its sister *Hanseniaspora* spp. that are are auxotrophic for thiamine [33, 45]. A closer look into the genes involved in thiamine biosynthesis in *S. ludwigii* UTAD17 revealed that this yeast encodes 10 enzymes required for conversion of histidine and pyridoxal-phosphate into the thiamine precursor hydroxymethylpyrimidine diphosphate (HMP-P), three enzymes for conversion of HMP-P into HMP-PP and four predicted thiamine transporters (Fig. 4). This is interesting since in *L. fermentati* and in *T. duelbreckii* we could only identify one enzyme for each of the different enzymatic steps required for biosynthesis of 3-HMP-PP, similar to what is reported for *Kluveromyces lactis*, *K. thermotolerans* or *Saccharomyces kluyveri* [46] (Fig. 4 and supplementary Table S6). In fact, until thus far the expansion of enzymes involved in synthesis of 3-HMP-PP has been described as a specific feature of the *Saccharomyces* sensu strictu species that harbor 3 enzymes for the synthesis of 3-HMP-P (Thi5, Thi11, Thi12 and Thi13) and two for the synthesis of 3-HMP-PP. [46] The amplification of only the 3-HMP-P branch, but not the HET branch (which provides the other precursor for thiamine biosynthesis; Fig. 4) is noteworthy (Fig. 4). 3-HMP-P has only been described as an intermediate of thiamine biosynthesis and thus it is not clear the outcome that might be obtained by *S. ludwigii* cells with the expansion of enzymes involved in synthesis of this precursor. In the case of *S. cerevisiae* the expansion of genes producing 3-HMP-P was proposed to assure proper channeling of pyridoxine to thiamine biosynthesis avoiding depletion in biosynthesis of aminoacids [46]. Genes required for catabolism of lactate, mannose, sucrose, raffinose and starch were also found in the genome of UTAD17 (Fig. 4, supplementary Table S6), consistent with the demonstrated ability of *S. ludwigii* UTAD17 and other strains of this species to grow on these sources (results not shown) [1]. As said above, *S. ludwigii* UTAD17 strain is not equipped with genes allowing catabolism of galactose and we could also not detect genes for catabolism of lactose, this observation being in line with the reported inability of this species to grow on these carbon sources [1].

Concerning nitrogen metabolism, all the genes required for synthesis of proteogenic amino acids, synthesis and degradation of GABA and for conversion of amino acids into higher alcohols through the Ehrlich pathway were found in the *S. ludwigii* UTAD17 ORFeome (supplementary Tables S6 and S7 and Fig. 4). No genes encoding enzymes for the synthesis of spermidine, spermine or putrescine, or for biosynthesis of methionine through the salvaging pathway (the main source of percursors for the biosynthesis of polyamines) were found in the genome of UTAD17, similar to what was observed for *Hanseniaspora* species [33, 45]. Although this observation is intriguing, considering that polyamines, and specially spermidine, plays a detrimental role in mediating growth in *S. cerevisiae* [47], it is in line with previous reports of the inability of the UTAD17 strain, and of *S. ludwigii* species in general, to produce biogenic amines (which are produced from polyamines) [48, 49]. Another noticeable difference between *S. ludwigii* UTAD17 and its sister species *Hanseniaspora* species was the observation that *S. ludwigii* is equipped with specific permeases for methionine, GABA, histidine, proline, glutamine, lysine, arginine, choline, isoleucine/valine/isoleucine, besides encoding five putative general amino acid permeases while *Hanseniaspora* encodes only two specific amino acid permeases but thirteen general amino acid permeases (Fig. 4 and supplementary Table S6) [33].

### The predicted FLAVOROMA genes in *S. ludwigii* UTAD17

One of the aspects for which *S. ludwigii* is considered to have strong biotechnological potential is its use in tailored flavour-fermented beverages [5, 26, 29, 48–50]. In this context, we searched the UTAD17 ORFeome for genes predicted to be involved in formation of volatile aroma compounds, with the more relevant aspects of this analysis being summarized in the metabolic map shown in Fig. 4 and further detailed in supplementary Table S7. *S. ludwigii* UTAD17 is equipped with enzymes for biosynthesis of ethyl esters, namely ethyl acetate (Fig. 4 and supplementary Table S7). In specific, we could identify two alcohol acetyl-transferases in the genome of the UTAD17 strain, *SCLUD4.g700*, moderately similar to *S. cerevisiae* Eat1 and *SCLUD6.g215*, weakly similar to the *Kluveromyces lactis* KlEat1 and *Wickerhamomyces anomalus* WaEat1 (Fig. 4 and supplementary Table S7). Eat1 from *W. anomalus* and *K. lactis* were recently described as part of a novel family of alcohol acetyltransferases essentially responsible for ethyl acetate production [51]. No orthologues of ScAtf1 or ScAtf2, two other alcohol acetyltransferases that in *S. cerevisiae* also contribute for synthesis of ethyl acetate, were identified in the ORFeome of *S. ludwigii* UTAD17. Similarly, no orthologues for ScAtf1 or ScAtf2 were found in the genome of *Hanseniaspora* species whose ability to produce ethyl esters (a trait for which these species are particularly known for –e.g. [4]), was hypothesized to result from the activity of a specific set of putative set alcohol acetyl transferases only found in the genomes of these species [33] and whose functional characterization is being pursued in our laboratory. Strikingly, although the UTAD17 strain is equipped with enzymes leading to synthesis of ethyl esters and *S. ludwigii* is known for high capability to produce ethyl esters [5, 28, 29, 52], during fermentation of natural grape juice the production of these volatiles by UTAD17 cells was almost negligible, even below the one exhibited by *S. cerevisiae* [48]. Further investigations will have to be performed to better understand this observation specially focusing whether this trait is specific of the UTAD17 strain (which by some reason could have the activity of alcohol acetyl-transferase enzymes impaired) or whether this resulted from the composition of the grape juice used in the fermentations that could be less favourable for production of ethyl esters by *S. ludwigii* cells.

*S. ludwigii* is also known for its ability to produce 2,3-butanediol and acetoine [5, 50, 53]. Consistently, three predicted 2,3-butanediol dehydrogenases (*SCLUD5.g30*, *SCLUD7.g344* and *SCLUD8.g438*) are found in the genome of the UTAD17 strain (Fig. 4 and supplementary Table S7). Enzymes for the production of higher alcohols, isoamyl alcohols and a putative β-glucosidase were also detected in the genome of the UTAD17 strain (supplementary Table S7).

The retention of aromatic compounds in wines (namely fruity esters) has been linked to increased content of mannoproteins, which have also been found to increase mouth feel, provide protection against protein and tartaric instability and reduced astringency [54, 55]. *S. ludwigii* is known for its enhanced ability to excrete mannoproteins [28, 48, 56]. Further characterization of these mannoproteins released by *S. ludwigii* showed a very high content (above 90%) of mannose suggesting that hyper-mannosylation of these proteins released from the cell wall occurs [56]. Notably, mining of the *S. ludwigii* UTAD17 genome allowed us to identify five putative β-mannosyltransferases (SludBMT1, SludBMT2, SludBMT3 and SludBMT4) with strong homology to β-mannosyltransferases described in *Candida albicans, C. glabrata* or *Pichia pastoris* [57, 58] (supplementary Table S8 and Fig. 4). In those species, these enzymes mediate the incorporation of β-1,2-linked oligomannosides in the cell wall (a unique feature since in *S. cerevisiae* these are α-1,4-linked) and to promote hyper-mannosylation of secreted proteins [57, 58]. No orthologues of these enzymes could be found in *H. uvarum* or in *H. guilliermondii*, while *H. osmophila* appears to encode only one of these mannosyltransferases (supplementary Table S8). The presence of these β-mannosyltransferases in the genome of *S. ludwigii* UTAD17 strongly suggests that the composition of the cell wall should be quite different from the one found in other *Saccharomycodeacea* and in *S. cerevisiae*, which can affect the function of this structure as a selective barrier (for example, against SO_2_, as it will be discussed below).

### Elucidating *S. ludwigii* stress responses relevant in the context of wine fermentation: emphasis on tolerance to sulfur dioxide

The fact that contamination by *S. ludwigii* is observed either in sulfited grape-musts or in stabilized wines [5, 9, 10] indicates that this yeast is equipped with means to survive the harsh enviroment of vinification which include, among others, the high concentration of sugars present in the beginiging of the fermentation or the high concentrations of ethanol obtained in the end, besides the presence of inhibitory concentrations of SO_2_. Although not much is known concerning how wine Non-*Saccharomyces* yeasts respond to environmental stress, a lot of knowledge was gathered on the field in *S. cerevisiae* including the genes necessary for tolerance to ethanol [59, 60], to high concentrations of glucose [61] or even those required for this species to thrive in oenologically relevant conditions (the so-called fermentome [62]). We have searched the genome of UTAD17 for orthologues of these sets of stress-tolerance genes described in *S. cerevisiae* and found that *S. ludwigii* UTAD17 harbors around 40–45% of those genes, as detailed in supplementary Table S9. This percentage is considerably higher than the one found in *H. uvarum* or *H. guilliiermondii* that only harbored 33% of the ethanol-tolerance genes and 35% of the “fermentome” genes [33]. A closer comparison revealed that *S. ludwigii* UTAD17 encodes all the “Sc fermentome”-genes identified in *Hanseniaspora* and also 37 additional others that were not found in *Hanseniaspora* (supplementary Table S9). *S. ludwigii* UTAD17 was also found to encode 226 *S. cerevisiae* ethanol-resistance genes that could not be identidied in its sister species *Hanseniaspora* (details in supplementary Table S9). Although these numbers need to be taken carefully because the proteins might have suffered divergent evolutive paths in *S. cerevisiae* and in the other yeasts and therefore the absence of an homologue does not necessarily mean that a functional orthologue is absent, the higher presence of stress genes in *S. ludwigii* UTAD17 is consistent with the described increased resilience of this species to wine-related stresses, compared with *Hanseniaspora* species. From the analysis performed it stood out that a large cohort of peroxissomal genes and mitochondrial proteins involved in translation and in the respiratory chain are present both in *S. cerevisiae* and in *S. ludwigii* but are absent from *Hanseniaspora* genomes (supplementary Table S6). Although the molecular mechanism by which the peroxissomal function contributes for tolerance to ethanol in *S. cerevisiae* could not be uncovered until thus far, it was clear that when cells are challenged with toxic concentrations of ethanol a proliferation of these organelles occurs [59], which is also consistent with the protective effect exerted by *PEX* genes [59, 60] and with their reported up-regulation under ethanol stress [63]. Further investigations will be required to confirm whether or not perixossomal function plays a role in mediating tolerance to ethanol in *S. ludwigii*.

*S. ludwigii* UTAD17 does not encode a clear orthologue for the transcription factors Msn2 and Msn4 [64], responsible for the control of the environmental stress response in *S. cerevisiae* and that also play a role in response of this species to vinification conditions [65, 66]. Closer mining of the *S. ludwigii* genome allowed us to identify one protein (*SCLUD3.g330*) that shows similarity at the level of the C-terminal domain (67% identity) to the C-terminus of ScMsn2 and ScMsn4, the region that comprises the DNA binding domain of these regulators [67] (supplementary Figure S4). Due to evolution transcription factors tend to conserve their homology across more distant species essentially at the DNA binding domain, while transactivation domains are largely more variable. In *Hanseniaspora* species no orthologue of ScMsn2/ScMsn4 [33] nor this was also found in the *Saccharomycetacea* species *S. pombe* or *S. kluyverii* [68]. This does not mean that *Hanseniaspora* or *S. ludwigii* do not mount an environmental stress response with some similarities to the one described in *S. cerevisiae* since such a response was described to occur in *S. pombe* or *S. kluyverii* [68], albeit the absence of a Msn2/Msn4 clear orthologue.

**Fig. 5:**
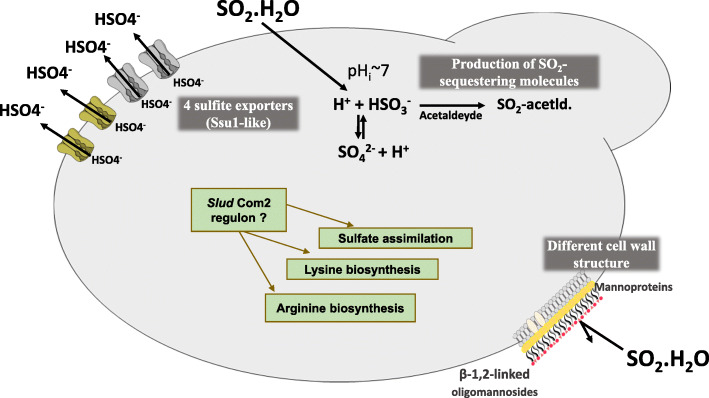
Mechanisms suggested to contribute for tolerance to SO_2_ in *S. ludwigii*, as suggested by mining the genome of the UTAD17 strain. The candidate players that might contribute for the enhanced tolerance to SO_2_ exhibited by *S. ludwigii* cells, as suggested by mining of the genome of the UTAD17 strain, were selected and are herein highlighted. Besides the four predicted sulfite exporters with similarity with the sulfite export pump Ssu1 from *S. cerevisiae*, orthologues for genes that been found to mediate tolerance to SO_2_ in *S. cerevisiae* such as genes involved in biosynthesis of lysine and arginine or the genes involved in the sulfate assimilation pathway, are also indicated. The eventual involvement of a putative Com2-regulatory pathway in *S. ludwigii* is also hypothesized, based on the existence of a transcription factor with some degree of similarity to this crucial SO_2_-determinant in *S. cerevisiae* (see details for further discussion in the text). It is also hypothesized whether the presumed different structure of the *S. ludwigii* cell wall, resulting from this species harboring a set of mannoproteins and a different structure of the β-glucan (compared to the one exhibited by in other *Saccharomycodeacea* species and by *Saccharomycetacea*) can contribute for the reported reduced diffusion of SO_2_ into the inside of *S. ludwigii* cells [19]. Further information about candidate SO_2_-tolerance genes in S. ludwigii is provided in supplementary Table S10. This schematic representation is original and was specifically prepared by the authors to be presented in this manuscript

Tolerance to SO_2_ in *S. cerevisiae* has been largely attributed to the activity of the sulfite export pump Ssu1 [21, 22, 69], which was also found to influence tolerance to this preservative in the more tolerant strain *Brettanomyces bruxellensis* [23]. Notably, the genome of *S. ludwigii* UTAD17 was found to encode four genes with a strong similarity (above 45% at the amino acid level) with the efflux pump ScSsu1: SCLUD1.g608, SCLUD1.g608b, SCLUD1.g612, SCLUD1.g612b) (supplementary figure S5). These four genes are arranged in tandem and appear as two duplicated pairs with *SCLUD1.g608* and *SCLUD1.g612* showing higher similarity to ScSsu1 (47.5% identity) and *SCLUD1.g608b*, *SCLUD1.g612b* showing a lower similarity due to a premature STOP codon that renders the proteins shorter at the C-terminal region (Fig. 5 and supplementary Figure S5). This is very interesting since it is the first time that a candidate sulfite export system is described in *S. ludwigii*. Our preliminary results from a transcriptomic analysis undertaken in SO_2_-challenged *S. ludwigii* cells confirms that the four *SSU1* genes are transcribed (albeit the shorter versions at a considerably lower extent than the larger ones) and their transcription is augmented upon exposure to SO_2_ (results not shown). Further studies will be required to investigate the individual role of these four “Ssu1-like” pumps in determining tolerance to SO_2_ in *S. ludwigii*. More SO_2_-tolerant *B. bruxellensis* strains were found to encode alleles with higher activity of *SSU1* than those encoded by less susceptible strains, this being attributed to the existence of point mutations that result in increased activity of the pumps [23]. In this sense, it will also be interesting to investigate if a similar trait is observed in the case of the *SSU1* genes encoded by *S. ludwigii* considering that a strong inter-strain variability concerning tolerance to SO_2_ has also been described [2]. In *S. cerevisiae* transcription of *SSU1* is largely dependent of the transcriptional activator Fzf1 [70] but we could not identify an orthologue for this regulator in the genome of *S. ludwigii* (nor in *B. bruxellensis*) suggesting that in this species the control of the sulfite efflux pump could be under the control of a different regulatory circuit.

Recently our group has performed a genome-wide phenotypic screening that identified around 200 genes required for tolerance to SO_2_ in *S. cerevisae* expanding the set of resistance determinants to this preservative well beyond Ssu1 [21]. Among these newly identified SO_2_-resistance genes was the Com2 transcription factor, an orphan homologue of Msn2, which was identified as being critical not only for tolerance but also for the reprogramming of *S. cerevisiae* transcriptome in response to SO_2_ stress [21]. Although the *SCLUD3.g330* regulator discussed above shows more homology with ScMsn2 than with ScCom2 (supplementary Figure S2), it still remains the question of whether or not this regulator could mediate response and/or tolerance of *S. ludwigii* to SO_2_. Around 65% of the other SO_2_-resistance genes identified in *S. cerevisiae* had a robust orthologue in the ORFeome of *S. ludwigii* UTAD17 including the genes mediating the sulfur assimilation pathway (e.g. *MET14*, *MET16* and *MET3*) or the genes involved in biosynthesis of lysine and arginine (Fig. 5 and suplementary Table S10).

Another aspect that might also influence the extreme tolerance to SO_2_ of *S. ludwigii* is the different structure of the cell wall which, as discussed above, is likely to be enriched in β-1,2-mannosides due to the presence of β-mannosyltransferases. This modification could result in a lower permeability of *S. ludwigii* cell wall to SO_2_ and thus explain the observed reduced diffusion rate into the inside of these cells, compared to the one observed for *S. cerevisiae* [19, 20]. Since ethanol tolerance has also been demonstrated to depend on diffusion across the cell wall, it is possible that this anticipated difference in the cell wall of *S. ludwigii* can also contribute for its higher tolerance to ethanol, specially when compared to its closely related *Hanseniaspora* species. In Fig. 5 we have schematically represented the features that might influence tolerance to SO_2_ in *S. ludwigii*, as uncovered by the herein described genomic analysis. What can be the individual contribution of these different players for the overall phenotype exhibited by this species and how they might determine intra-strain variability, is something that will need future studies focused on the herein uncovered candidates.

## Conclusions

In this work we have deepened the genomic sequence and annotation of the wine spoilage species *S. ludwigii* UTAD17 shedding light into relevant aspects of the biology and physiology of this species such as its high resilience to the wine preservative SO_2_ or its resilience to thrive in the challenging environment of the wine must. Compared to its sister *Hanseniaspora* species, that also belong to the *Saccharomycodeacea* family, significant differences were observed including functional neoglucogenesis, glyoxylate and thiamine pathways or a different cell wall structure. We have also unravelled aspects that might render a suitable exploration of *S. ludwigii* as an interesting microbial cell factory either as a co-adjuvant in production of fermentation beverages, or in production of aroma compounds (e.g. ethyl acetate) or of biofuels (e.g. production of bioethanol using the N-GlcNAc enriched renewable source chitin).

## Methods

### Strain

The autochthonous *Saccharomycodes ludwigii* UTAD17 strain, part of the collection of wine strains owned by the UTAD laboratory, was isolated in a wine must obtained with grapes harvested from the Douro demarcated region harvested from the experimental vineyard of UTAD (with approved use for research), in Portugal [24, 48]. Presumptive identification of UTAD17 as a non-*Saccharomyces* strain was based on the ability of these cells to grow on L-lysine agar selective medium, followed by microscopic examination of the size and morphology of the colonies. Further confirmation of the identity of UTAD17 as belonging to the *S. ludwigii* species was performed by Sanger sequencing of the conserved D1/D2 ribosomal region using the NL1 (5′-GCATATCAATAAGCGGAGGAAAAG-3′) and NL4 (5′-GGTCCGTGTTTCAAGACGG-3′) primers which showed more than 99.9% identity with ribosomal sequences reported from other *S. ludwigii* strains.

### Pulsed-field gel electrophoresis (PFGE)

Separation of *S. ludwigii* UTAD17 chromosomal DNA was carried out as described by Sipiczki et al. [71] and as modified by El Hage & Houseley [72]. Briefly, yeast chromosomes were separated in 1% agarose gels in 0.5 x TBE buffer cooled at 12 °C in a BioRad CHEF-DRIII electrophoresis apparatus (Bio-Rad, Hercules, CA, USA). Electrophoresis was conducted at 3 V/cm for 36 h with a 200–300 s ramping switch interval and for 60 h with a 300–600 s ramping switch interval. The CHEF-DNA size markers used to calculate the molecular sizes of UTAD17 chromosomal bands were *Hansenula wingei*, for chromosome bands ranging from 1.05 to 3.13 Mbp and *S. cerevisiae* (for chromosomes below 1.05 Mbp). The molecular sizes for *S. ludwigii* UTAD17 chromosomes were then calculated through a calibration curve (band distance vs molecular size) making use of ImageJ software.

### Genome sequencing, assembly and annotation of *S. ludwigii* UTAD17

Genomic DNA extraction of *S. ludwigii* UTAD17, as well as subsequent sequencing and assembly was performed as a paid service at CD Genomics (Shirley, New York, United States). Briefly, genomic DNA of *S. ludwigii* UTAD17 was extracted using Quiagen Magattract HMW kit according to the manufacturer’s instructions. The DNA quality was evaluated using a Qubit fluorometer and a Fragment Analyzer™ Automated CE System combined High Sensitivity Large Fragment 50Kb Analysis Kit. Qualified genomic DNA was fragmented using Covaris g-TUBE devices and were subsequently repaired by treating the sample with a DNA-damage repair mix. Following DNA-damage repair, blunt ends were created on each end and then hairpin adapters incorporating a unique barcode were ligated to each blunt end. The SMRTbell DNA template libraries were selected using a bluepippin system targeting a fragment size > 10 kb. Library quality was analyzed by Qubit and average fragment size was estimated using a Agilent 2100 Bioanalyzer. We used Sequel Sequencing kit 2.1 to sequence the library in PacBio Sequel platform. For the bioinformatics analysis, we first demultiplexed the PacBio subread file with Lima package, after which the demultiplexed bam file was converted to FASTA format using SAMtools FASTA. Flye was used to assemble the FASTA file with “--plasmids --iterations 2 --asm-coverage 120” parameters. The completeness of the genomics data was assessed using BUSCO11 (version 4.1.2, run in mode genome and proteome with the lineage dataset: saccharomycetes_odb10). 91% was obtained when the software was run in the genome mode and 96% when run in the proteome mode. The results obtained with BUSCO allowed us to estimate the degree of reads contamination as being below 1%. To further curate the PacBio-assembled contigs and avoid sequencing mistakes the reads obtained from prior sequencing by Illumina of UTAD17 genome (Tavares et al., 2017) were mapped in the 20 contigs obtained (resulting in more than 95% mapping). The annotation of the 20 curated PacBio contigs was performed in the Geneious software framework (version 2019.2.3). First we started by mapping in the contigs the previously determined ORFs of UTAD17 [24]. Afterwards the ab initio gene detection algorithm Augusts (trained in *S. cerevisiae*, *S. pombe* and *A. nidulans*) was used to identify putative CDSs in the sequence of the contigs. Whenever the predicted gene models coincided with CDSs previously described as belonging to the *S. ludwigii* UTAD17 ORFeome these were automatically validated. BLASTP analysis using the UNIPROT database as a target was used to curate and modify the gene models predicted in silico. Those gene models having an identified hit at UNIPROT were considered valid while those that didn’t comply with this criterion were considered hypothetical. To obtain further information concerning the annotation, including functional categorization, the OmicsBox (version 1.1.164) framework was used.

### Metabolic reconstruction and comparative proteomic analysis of S. ludwigii UTAD17 ORFeome and other yeast species

Metabolic reconstruction of *S.ludwigii* UTAD17 was performed making use of KEGG BlastKoala annotation tool [44] using as a query dataset the 5008 genes predicted in the in silico annotation, choosing Fungi as the taxonomic group and enabling Koala to search against the family_eukaryotes.pep KEGG database. To further improve this functional annotation the eggNOG-mapper [34] set at the default parameters was also used. For the comparative analysis of the *S. ludwigii* UTAD17 ORFeome with the proteomes of *S. cerevisiae* EC1118, *H. guilliermondii* UTAD222, *H. uvarum* AWRI3580, *H. osmophila* AWRI3579, *T. delbrueckii* CBS1146, and *L. fermentati* CBS6772 pairwise BLASTP analyses were performed using the sets of proteins publicly available at UNIPROT for each strain. Two proteins from the different yeast species under analysis were considered highly similar whenever identity associated with the pairwise alignments was above 50% had an associated *e*-value below e^− 50^. Whenever protein pairwise alignments resulted in identities between 30 and 50% with an associated e-value below e^− 20^, the corresponding proteins were considered similar. In all the other cases the protein pairs were considered dissimilar. In order to assess genetic relatedness between the different strains used in the comparative proteomic analysis the IST sequence of these strains (and also of others belonging to the same species and available at NCBI) was aligned using MUSCLE (Edgar et al. 2014) and used for phylogenetic distance analysis on MEGA X (Tamura et al. 2013). For this, the maximum likelihood method was used and general time reversible model chosen based on Neighbor-Join and BioNJ algorithms applied to a matrix of pairwise distances estimated using the Maximum Composite Likelihood (MCL) approach. The phylogenetic analysis was performed using default parameters, and a bootstrap method analysis with 250 replications. The ITS sequence of *Schizosaccharomyces pombe* 972 h was used as an outgroup.

## Supplementary Information

**Additional file 1 **Supplementary figures – This file has included a set of 5 supplementary figures providing: a dendrogram comparing the ITS region of the yeast strains used for the comparative proteomic analysis performed (**Figure S1**), phographs obtained by microscopic imaging of S. ludwigii UTAD17 cells cultivated in minimal medium having glucose or GlcNAc as the sole carbon source (**Figure S2**), a functional distribution of the *S. ludwigii* UTAD17 ORFeome, as performed by the BLASTKoala Annotation tool (**Figure S3**), the alignment of the protein sequence of the transcription factors ScMsn2, ScCom2 and the S. ludwigii predicted regulator *SCLUD3.g33* (**Figure S4**), the alignment of the protein sequence of the efflux pump ScSsu1 and of the four predicted *S. ludwigii* orthologues SCLUD1.g608, SCLUD1.g612, SCLUD1.g608b, SCLUD1.g612b (**Figure S5**).

**Additional file 2 **Supplementary tables This file has included a set of 10 supplementary tables providing: the list of *S. ludwigii* genes uncovered in this study but in the prior assembly (**Table S1**), the distribution of S. ludwigii genes across the 20 assembled contigs (**Table S2**), the functional clustering performed on the annotated S. ludwigii genes (**Table S3**), the list of *S. ludwigii* UTAD17 genes homologues for the 551 identified *S. cerevisiae* ohnologues (**Table S4**), the list of *S. ludwigii* UTAD17 genes for which we could not find orthologues in the genomes of *H. uvarum*, *H. guilliermondii*, *S. cerevisiae*, *T. delbrueckii* or *L. fermentati* (**Table S5**), functional annotation of genes involved in main carbon and nitrogen metabolic pathways in *S. ludwigii* UTAD17 and corresponding orthologues in *H. uvarum*, *H. guilliermondii*, *T. delbrueckii* or *L. fermentati* (**Table S6**); the list of genes predicted to encode enzymes influencing wine aroma (**Table S7**); the list of genes predicted to encode β-mannosyltransferases in the genome of *S. ludwigii* UTAD17 (**Table S8**); list of *S. ludwigii* UTAD17 genes orthologous to those found to mediate tolerance to ethanol, high glucose or to vinification conditions in *S. cerevisiae* (**Table S9**); list of *S. ludwigii* UTAD17 genes orthologus to those found to mediate tolerance to SO_2_ in *S. cerevisiae* (**Table S10**).

**Additional file 3 **Original PFGE gel used in the karyotyping of the *S. ludwigii* UTAD17 strain (original image from the one shown in Fig. 1).

## Data Availability

The sequence of the assembled 20 contigs and the annotation performed were submitted at NCBI (Bioproject PRJNA542099; Biosample SAMN11609663) and at NCBI nucleotide (VANJ00000000.1).

## Abbreviations

NSY: Non-Saccharomyces Yeast
SO_2_: Sulfur dioxide
ORF: Open Reading Frame
