# Supplementary material for: Genome sequencing, annotation and exploration of the SO2-tolerant non-conventional yeast Saccharomycodes ludwigii
Source: BMC Genomics. 2021 Feb 23;22:131. doi: 10.1186/s12864-021-07438-z (PMC7903802; doi:10.1186/s12864-021-07438-z)

Figure S1 – Dendogram of the alignment obtained for the Internal transcribed spacer (ITS) of different yeast strains belonging to the *Saccharomycodeacea* and *Saccharomycetacea* families, including the strains that were used for the comparative proteomic analysis described in Fig.3 (underlined in red). The ITS sequences of the strains were retrieved directly from the NCBI database or in some cases from available genomic sequences.

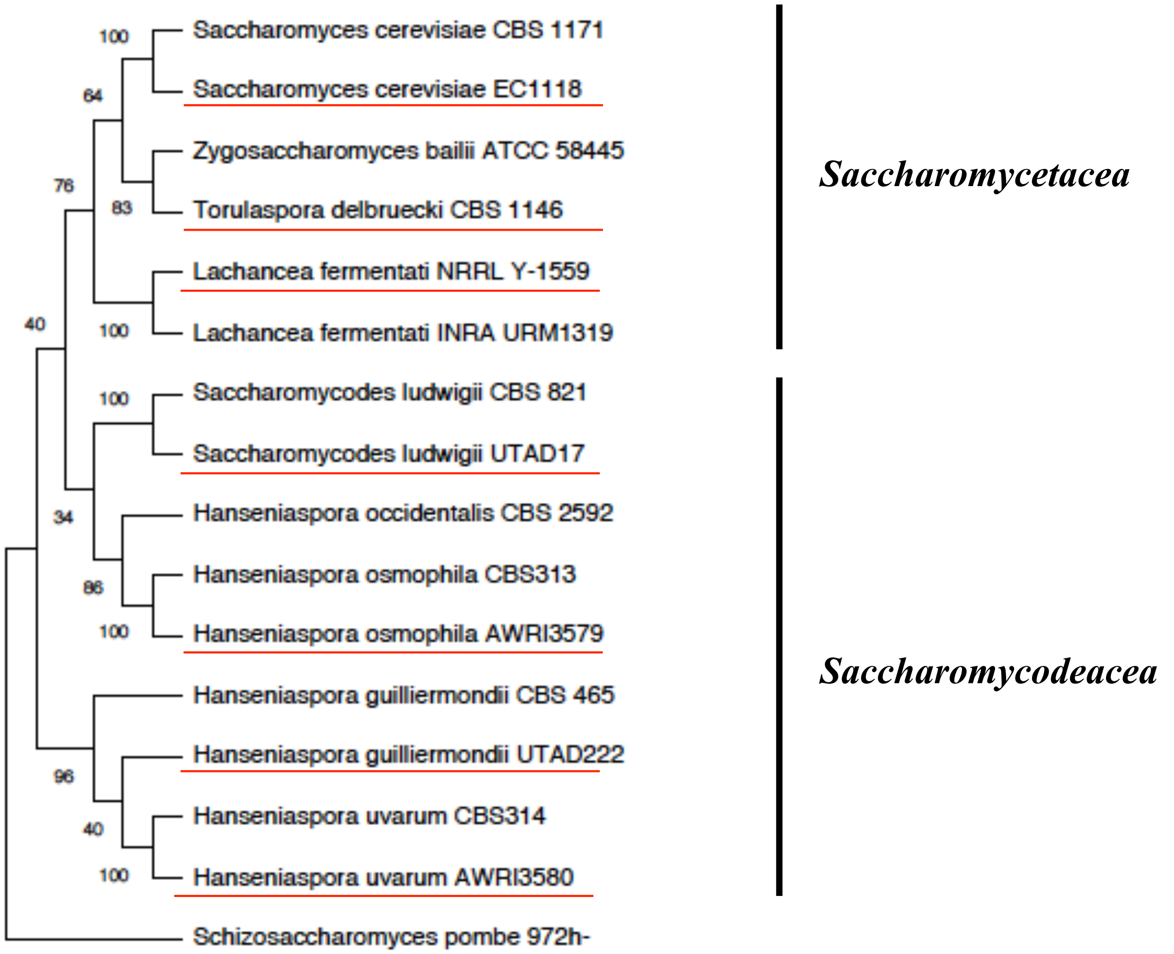

Figure S2 – Optic microscopy photographs of S. ludwigii UTAD17 during growth in minimal medium supplemented with glucose (2%) or GlcNAc (2%) as the sole carbon sources. Both images were obtained with a 40x objective after 6h of cultivation, at 30ºC and with 250 rpm, in the selected growth media. The images demonstrate the absence of filamentous growth.

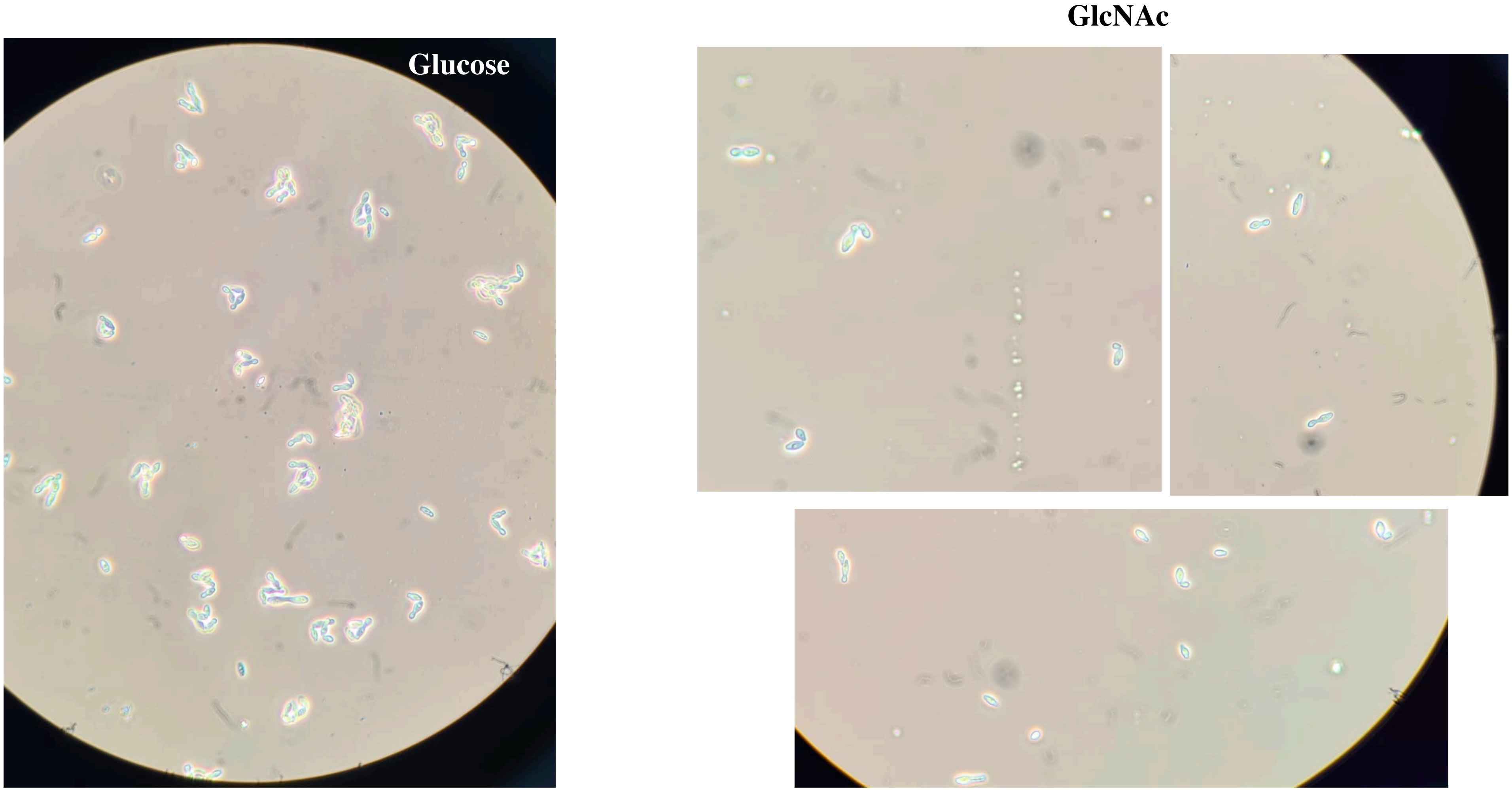

Figure S3 – Functional analysis of *S. ludwigii* UTAD17 as indicated by the metabolic reconstruction tool BLASTkoala. The number of *S. ludwigii* UTAD17 genes clustered in each of the functional categories is indicated inside the pie chart.

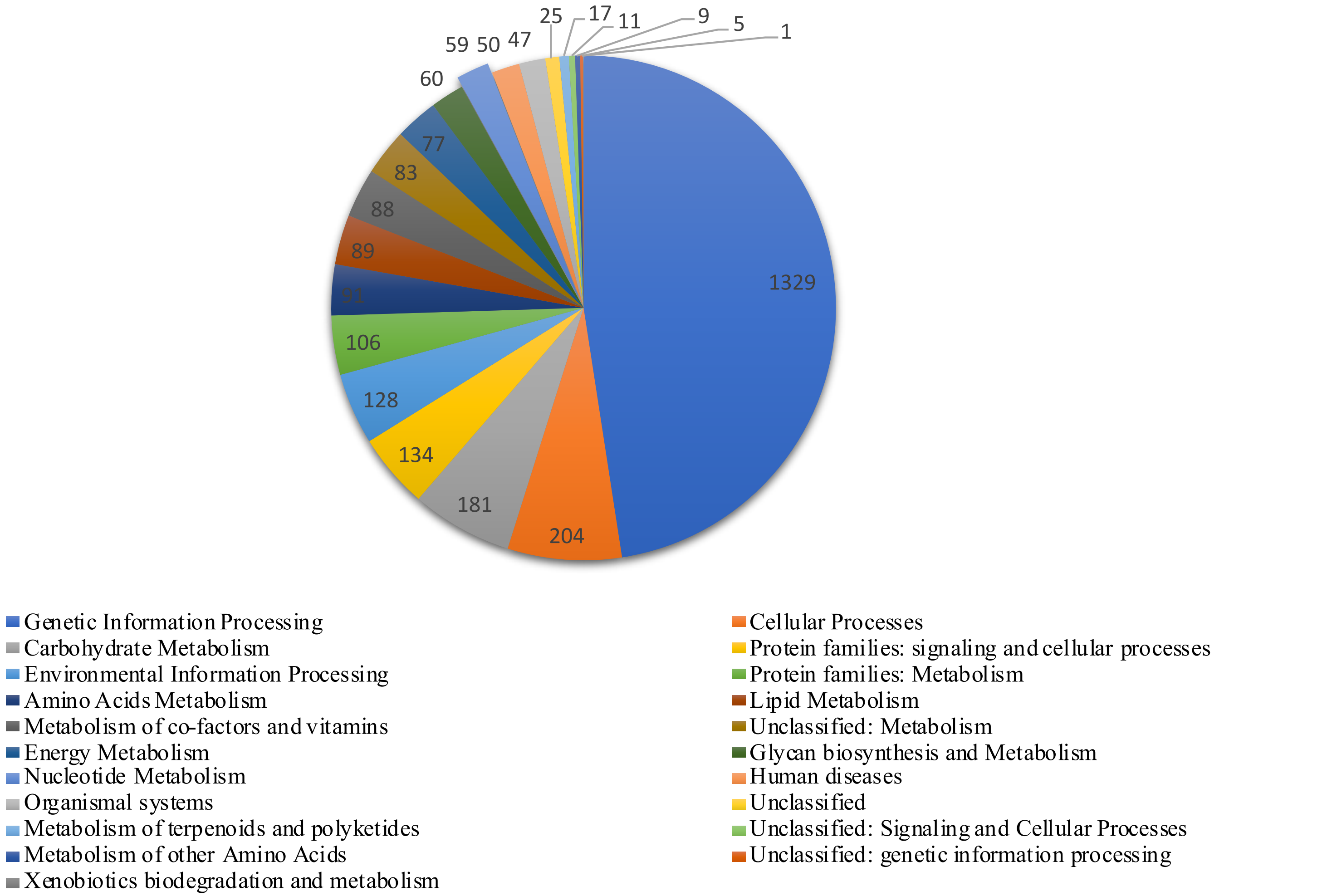

Figure S4 – Comparison of the amino acid sequence of the transcription factors ScCom2, ScMsn2 and SlMsn2 (ORF *SCLUD3.g330*). Using the available amino acid sequences for the different proteins a multiBLASTP analysis was undertaken using "Geneious Multiple Sequence Alignment ClustalW". Conserved residues are indicated in black boxes, being clear the highest degree of similarity at the C-terminal domain of the proteins which are known to harbor the DNA-binding domain of ScMsn2.

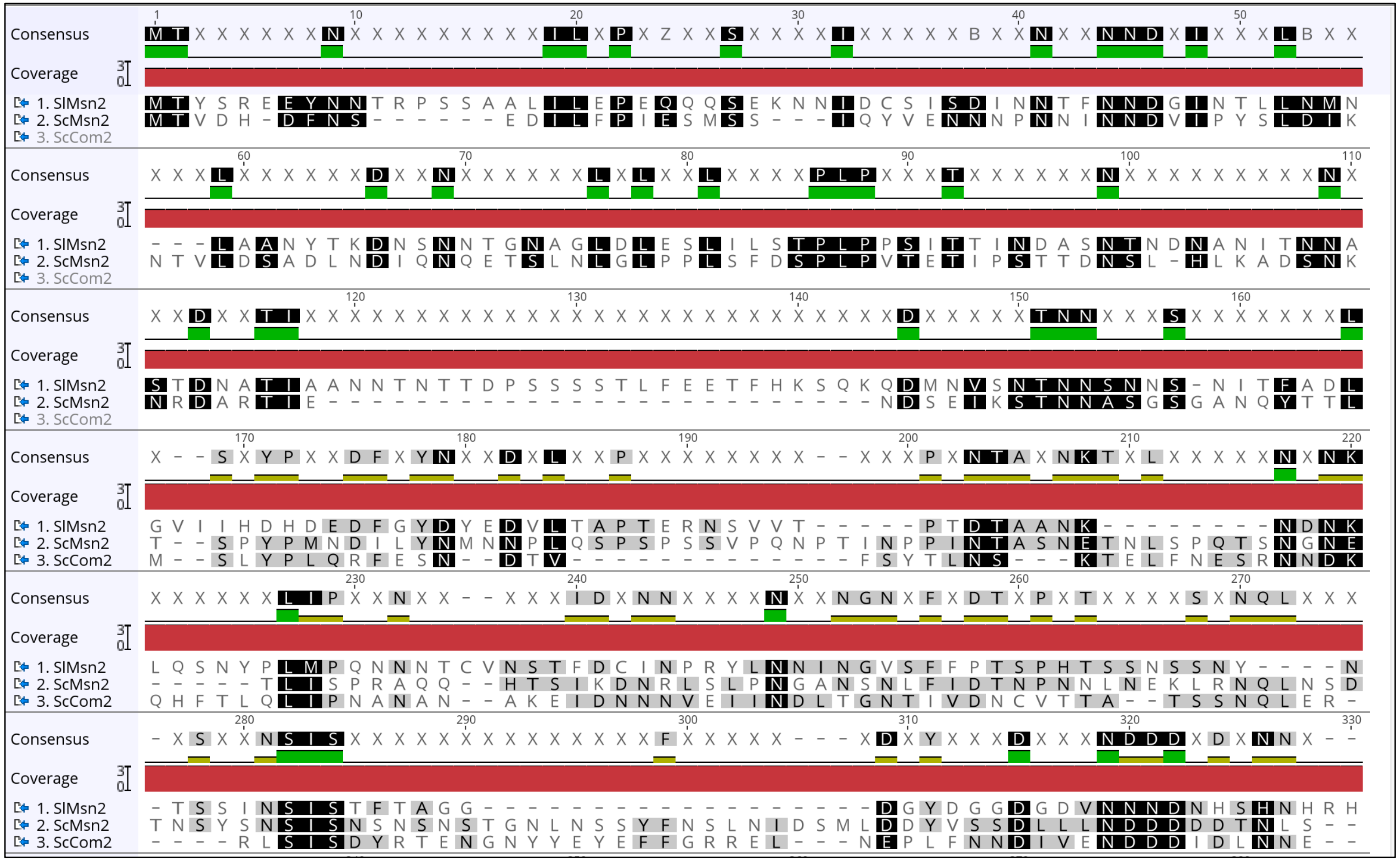

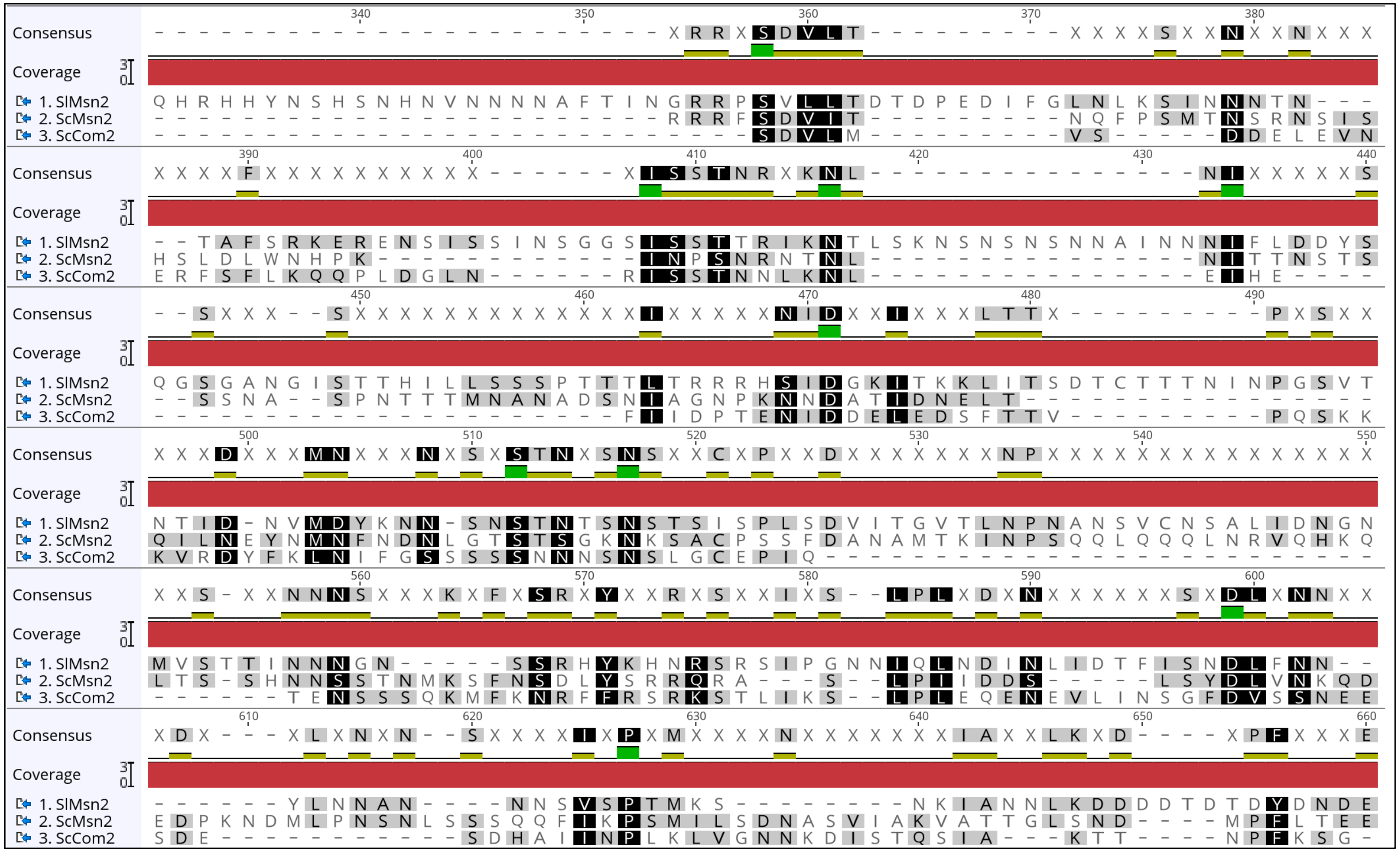
340 350 360 370 380
Consensus ------------------------XRRXSDVLT---------XXXXSXXNXXNXXX
Coverage 3 0
1. SlMsn2 QHRHHYNSHSNHNVNNNNAFTINGRRPSVLLTDTDPEDIFGLNLKSINNNTN---
2. ScMsn2 -----------------------RRRFSDVIT---------NQFPSMTNSRNSIS
3. ScCom2 ---------------------------SDVLM---------VS-----DDELEVN
390 400 410 420 430 440
Consensus XXXXFXXXXXXXXXX------XISSTNRXKNL---------------NIXXXXXS
Coverage 3 0
1. SlMsn2 --TAFSRKERENSISSINSGGSISSTTRIKNTLSKNSNSNSNNAINNNIFLDDYS
2. ScMsn2 HSLDLWNHPK------------INPSNRNTNL---------------NITTNSTS
3. ScCom2 ERFSFLKQQPLDGLN------RISSTNNLKNL---------------EIHE----
450 460 470 480 490
Consensus --SXXX--SXXXXXXXXXXXXXIXXXXXNIDXXIXXXLTTX----------PXSXX
Coverage 3 0
1. SlMsn2 QGSGANGISTTHILLSSSPTTTLTRRRHSIDGKITKKLITSDTCTTTNINPGSVT
2. ScMsn2 --SSNA--SPNTTTMNANADSNIAGNPKNNDATIDNELT----------------
3. ScCom2 ---------------------FIIDPTENIDDELEDSFTTV---------PQSKK
500 510 520 530 540 550
Consensus XXXDXXXMNXXXNXSXSTNXSNSXXCXPXXDXXXXXXXXNPXXXXXXXXXXXXXX
Coverage 3 0
1. SlMsn2 NTID-NVMDYKNN-SNSTNTSNSTSISPLSDVITGVTLNPNANSVCNSALIDNGN
2. ScMsn2 QILNEYNMNFNDNLGTSTSGKNKSACPSSFDANAMTKINPSQQLQQQLNRVQHKQ
3. ScCom2 KVRDYFKLNIFGSSSSSNNNSNSLGCEPIQ-------------------------
560 570 580 590 600
Consensus XXS-XXNNNSXXXKXFXSRXYXXRXSXXIXS--LPLXDXNXXXXXXSXDLXNNXX
Coverage 3 0
1. SlMsn2 MVSTTINNNGN-----SSRHYKHNRSRSIPGNNIQLNDINLIDTFISNDLFNN--
2. ScMsn2 LTS-SHNNSSTNMKSFNSDLYSRRQRA---S--LPIIDDS-----LSYDLVNKQD
3. ScCom2 ------TENSSSQKMFKNRFFRSRKSTLIKS--LPLEQENEVLINSGFDVSSNEE
610 620 630 640 650 660
Consensus XDX---XLXNXN--SXXXXIXPXMXXXXNXXXXXXXIAXXLKXD----XPFXXXE
Coverage 3 0
1. SlMsn2 ------YLNNAN----NNSVSPTMKS--------NKIANNLKDDDDTDTDYDNDE
2. ScMsn2 EDPKNDMLPNSNLSSSQQFIKPSMILSDNASVIAKVATTGLSND----MPFLTEE
3. ScCom2 SDE-----------SDHAIINPLKLVGNNKDISTQSIA---KTT----NPFKSG-

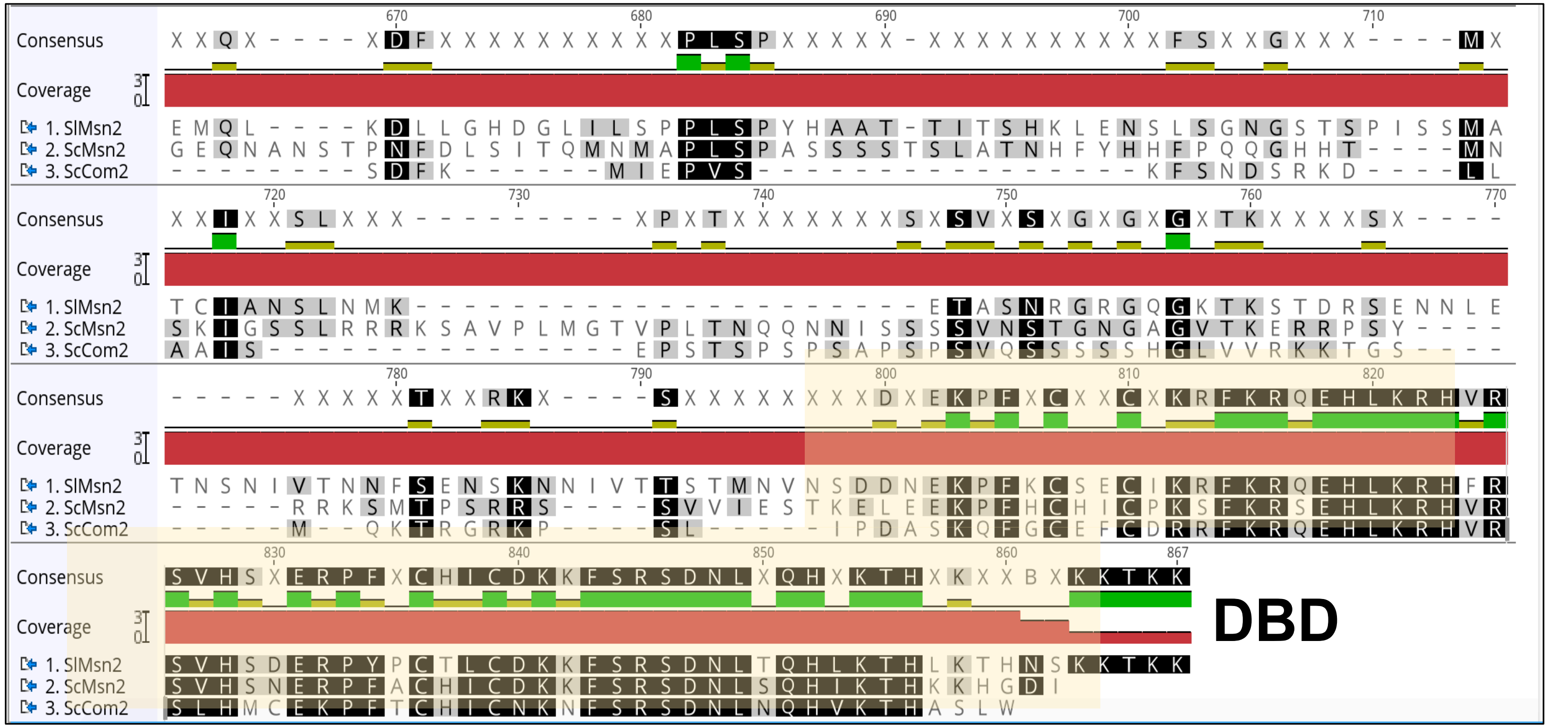
Consensus
Coverage
1. SlMsn2
2. ScMsn2
3. ScCom2
670
680
690
700
710
720
730
740
750
760
770
780
790
800
810
820
830
840
850
860
867
DBD

Figure S5 – Amino acid sequence alignment of the sulfite efflux pumps *Ssu1* of *S. cerevisiae* and the four predicted orthologues in *S. ludwigii* UTAD17. This alignment was performed with "Geneious Multiple Sequence Alignment ClustalW" using the amino acid sequences ScSsu1, SCLUD1.g608, SCLUD1.g612, SCLUD1.g608b, SCLUD1.g612b. The red arrow indicates the premature STOP codon found in the predicted sequences of two of the *S. ludwigii* UTAD17 orthologues.

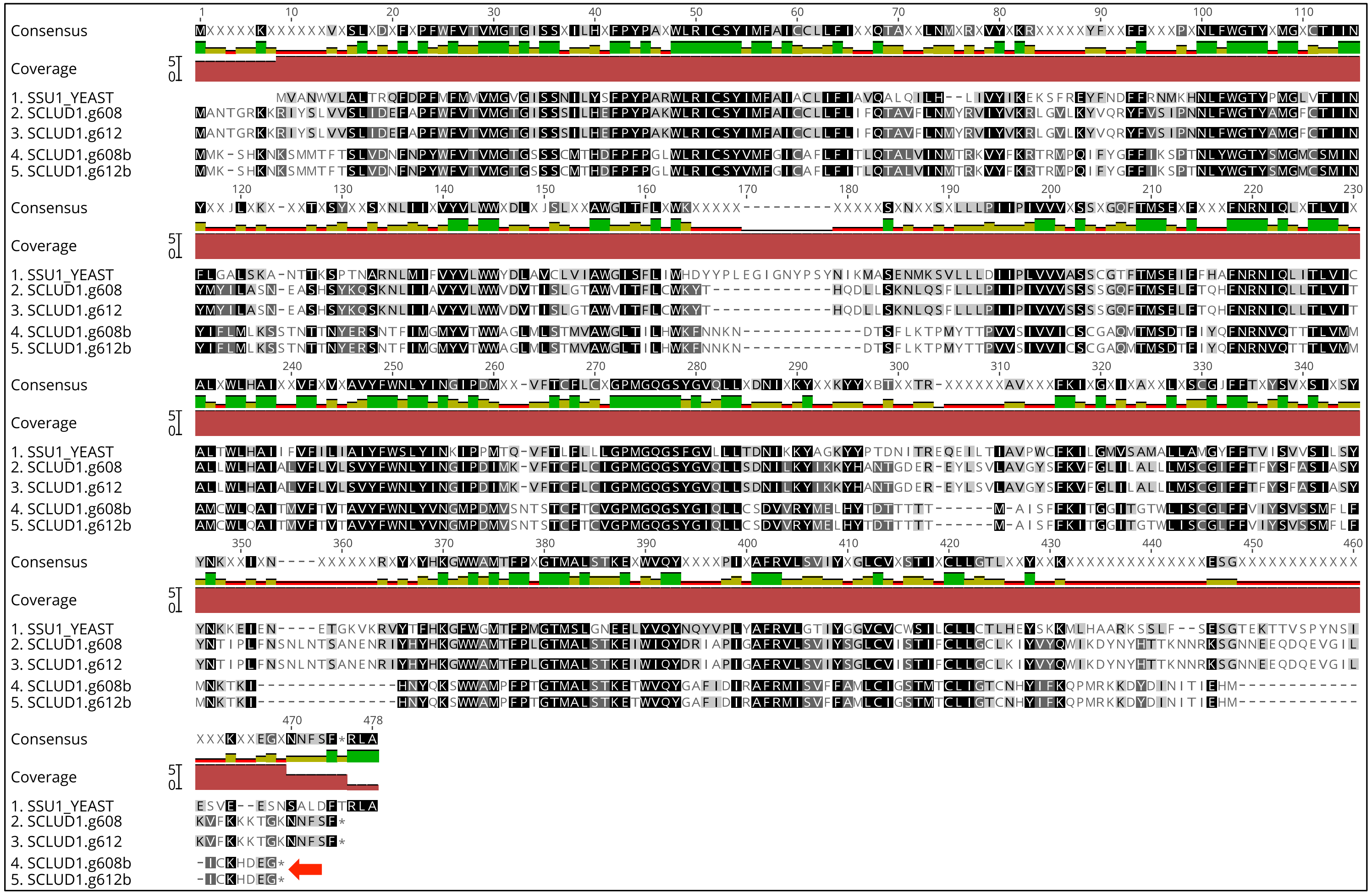

Supplement: Supplementary file 1 — Additional file 1 Supplementary figures – This file has included a set of 5 supplementary figures providing: a dendrogram comparing the ITS region of the yeast strains used for the comparative proteomic analysis performed (Figure S1), phographs obtained by microscopic imaging of S. ludwigii UTAD17 cells cultivated in minimal medium having glucose or GlcNAc as the sole carbon source (Figure S2), a functional distribution of the S. ludwigii UTAD17 ORFeome, as performed by the BLASTKoala Annotation tool (Figure S3), the alignment of the protein sequence of the transcription factors ScMsn2, ScCom2 and the S. ludwigii predicted regulator SCLUD3.g33 (Figure S4), the alignment of the protein sequence of the efflux pump ScSsu1 and of the four predicted S. ludwigii orthologues SCLUD1.g608, SCLUD1.g612, SCLUD1.g608b, SCLUD1.g612b (Figure S5). [file 12864_2021_7438_MOESM1_ESM.pdf]
