# Supplementary material for: Genome sequencing, annotation and exploration of the SO2-tolerant non-conventional yeast Saccharomycodes ludwigii
Source: BMC Genomics. 2021 Feb 23;22:131. doi: 10.1186/s12864-021-07438-z (PMC7903802; doi:10.1186/s12864-021-07438-z)
Supplement: Supplementary file 3 — Additional file 3 Original PFGE gel used in the karyotyping of the S. ludwigii UTAD17 strain (original image from the one shown in Fig. 1). [file 12864_2021_7438_MOESM3_ESM.pdf]

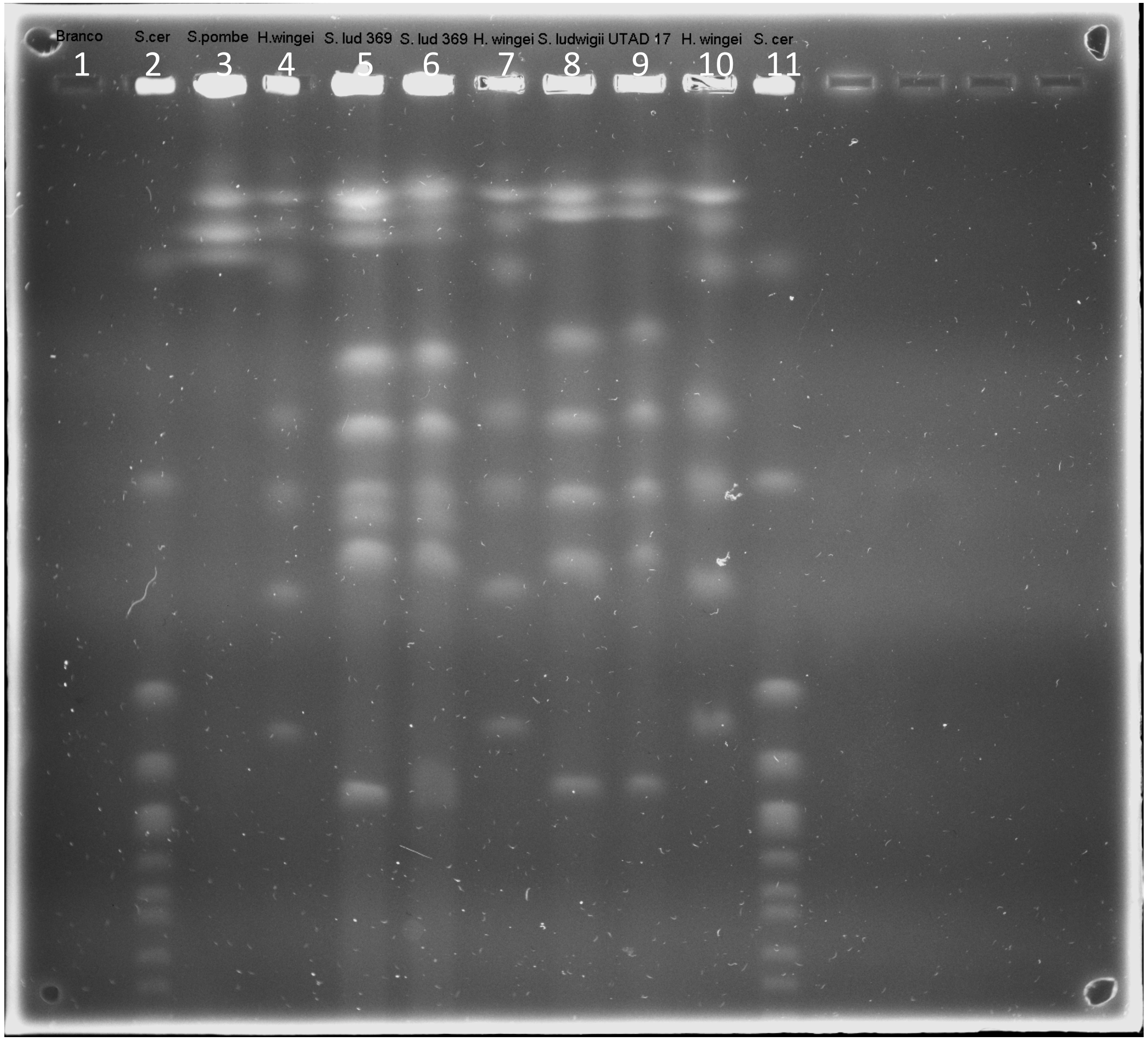

1- Blank
2 – *S. cerevisiae*
3 – Yeast A
4 – *Hansenula wingeii*
5 – Yeast *B*
6 – Yeast *C*
7 – *Hansenula wingeii*
8 – *S. ludwigii* UTAD17
9 – *S. ludwigii* UTAD17
10 – *Hansenula wingeii*
11 – *S. cerevisiae*

Karyotyping, by PFGE, of the genomic DNA of a series of yeast species including S. ludwigii UTAD17 (the strain under analysis in this work), *H. wingeii* and *S. cerevisiae* BY4741 (whose chromosomal bands were used as markers)
